# Dual β-lactam combination therapy for multi-drug resistant *Pseudomonas aeruginosa* infection: enhanced efficacy *in vivo* and comparison with monotherapies of penicillin-binding protein inhibition

**DOI:** 10.1038/s41598-019-45550-z

**Published:** 2019-06-24

**Authors:** Thanyaluck Siriyong, Rachael M. Murray, Lucy E. Bidgood, Simon A. Young, Florence Wright, Benjamin J. Parcell, Supayang Piyawan Voravuthikunchai, Peter J. Coote

**Affiliations:** 1 Biomedical Sciences Research Complex, School of Biology, University of St Andrews, The North Haugh, St Andrews, Fife KY16 9ST UK; 2 Present Address: Biosciences, Geoffrey Pope Building, University of Exeter, Stocker Road, Exeter, EX4 4QD UK; 3 Present Address: Faculty of Traditional Thai Medicine and Natural Product Research Center of Excellence, Prince of Songkla University, Songkhla, Thailand; 4 NHS Tayside, Medical Microbiology, Ninewells Hospital and Medical School, Dundee, DD1 9SY UK; 5 Department of Microbiology, Faculty of Science and Natural Product Research Center of Excellence, Prince of Songkla University, Songkhla, Thailand

**Keywords:** Antibiotics, Experimental models of disease, Pathogens

## Abstract

The aim of the study was to determine the efficacy of dual β-lactam combination treatments derived from eight approved drugs against *Galleria mellonella* larvae infected with MDR strains of *P*. *aeruginosa*. Carbapenem-resistant *P*. *aeruginosa* NCTC 13437 and an unrelated clinical isolate were used to infect *G*. *mellonella* larvae and the efficacy of twenty-eight dual β-lactam combination therapies were compared to their constituent monotherapies. For the most potent combinations identified, penicillin-binding protein (PBP) inhibition profiles were measured and compared with each constituent antibiotic. Five of the dual β-lactam combinations resulted in greater than 70% survival of infected *G*. *mellonella*. Two combinations showed potent, enhanced efficacy versus both strains − ceftazidime + meropenem and aztreonam + meropenem. Comparison of PBP inhibition profiles revealed that the enhanced efficacy of these two dual β-lactam combinations could not be explained by more potent inhibition of PBPs or inhibition of a broader range of PBPs. A possible contribution to the enhanced efficacy of the combinations could be stimulation of innate immunity via increased haemocyte numbers compared to their constituent monotherapies. Combinations of β-lactam antibiotics show promise in overcoming MDR *P*. *aeruginosa* and are worthy of additional study and development.

## Introduction

*Pseudomonas aeruginosa* infections in healthcare settings include pneumonia, urinary tract infections and surgical site infections. Risk factors include immunosuppression, prior antibiotic exposure, catheterisation and mechanical ventilation. Infections due to *P*. aeruginosa can result in bacteraemia and are associated with high mortality^1^. Effective treatment of *P*. *aeruginosa* infections can be problematic because the organism is intrinsically resistant, and readily acquires resistance, to multiple antibiotics. The incidence of isolation of multi-drug resistant (MDR) strains (defined as resistant to three or more classes of antibiotics) is increasing^2^.

Many antipseudomonal drugs belong to the β-lactam class of antibiotics and antipseudomonal β-lactams are essential for the successful treatment of *P*. *aeruginosa* infections. Resistant phenotypes often emerge during treatment via the selection of various complex chromosomal mutations. Frequently reported mutations include those leading to inactivation of the porin OprD, which decreases the drug permeability of the bacterial membrane, or those leading to de-repression of the intrinsic AmpC enzyme, a β-lactamase capable of hydrolysing penicillins and cephalosporins^3^. Other resistance-conferring mutations include those leading to up-regulation of drug efflux pumps^4^. Resistance can also result from the acquisition of transferable resistance genes such as those encoding extended-spectrum β-lactamases (ESBLs) or carbapenemases. In recent years, an increasing number of carbapenemase-producing *P*. *aeruginosa* outbreaks have been reported^5,6^. Worryingly, several of these have included metallo-β-lactamase (MBL) producing strains that are capable of rapidly hydrolysing carbapenems, a ‘last-resort’ treatment option for MDR Gram-negative infections^7–10^. MBL production is associated with higher mortality rates for *P*. *aeruginosa* infections^11^.

To try to circumvent this problem, many resistant *P*. *aeruginosa* infections are treated with dual combinations of antibiotics. The reasoning being that the simultaneous administration of two antibiotics with different modes of action increases the likelihood that the pathogen will be inhibited by at least one of the component drugs^12^. Receiving inappropriate initial antibiotic therapy correlates with patient mortality^13^, meaning that combination therapy can potentially improve initial therapy. Indeed, the use of combination therapy as an initial treatment has been shown in some cases to improve mortality rates for *P*. *aeruginosa* bacteraemia and pneumonia^14^.Traditionally, antipseudomonal combination therapy consists of a β-lactam plus a quinolone or an aminoglycoside. Clinical data supporting the use of β-lactams in combination with quinolones is lacking as most studies have found no difference in treatment outcomes between monotherapy and combination therapy^12^. There is more data to support the efficacy of β-lactams and aminoglycosides^15^, however this combination is associated with toxic side effects^16^ such as kidney damage^17^. The evidence for combination therapy providing real therapeutic benefit for *P*. *aeruginosa* infections remains contentious and data from a number of studies are conflicting (reviewed in^18^).

It is clear that novel treatments for MDR *P*. *aeruginosa* are required urgently as no new anti-pseudomonal antibiotics are likely to become available in the near future. Therefore, making optimal use of the antibiotics that are currently available in the form of novel combinations remains a realistic solution. Dual β-lactam therapy is an underexplored treatment option for MDR *P*. *aeruginosa* infections. Many *in vitro* studies were published in the 1980s that revealed extensive synergies between many different β-lactams against *P*. *aeruginosa*. *In vivo* studies, either in animal models or with human patients, were fewer but some also showed promise (extensively reviewed in^19^). None of these dual β-lactam combinations were developed further because at this time there was little clinical need due to lower incidence of MDR pathogens and the successful administration of antibiotic monotherapies. However, in light of the current aforementioned problems with treating MDR *P*. *aeruginosa*, re-exploring the potential of β-lactam combinations could prove beneficial.

The aim of this study was to evaluate the efficacy of twenty-eight dual β-lactam combinations derived from eight approved drugs that each represented a major class of the β-lactams. Efficacy of each combination therapy *in vivo* was compared with their constituent monotherapies against *Galleria mellonella* larvae infected with antibiotic-resistant strains of *P*. *aeruginosa* to identify combinations that could provide realistic future therapeutic options. For the most potent combinations identified, inhibition of penicillin-binding proteins (PBP) was compared to monotherapies to better understand the inhibitory action.

## Methods

### Bacteria and growth media

Three strains were used: *P*. *aeruginosa* NCTC13437, a characterised MDR strain (carrying the VEB-1 ESBL and VIM-10 MBL), that is resistant to carbapenems and other β-lactam antibiotics and also quinolones and aminoglycosides by unknown mechanisms^20^. A clinical strain of *P*. *aeruginosa* isolated from the sputum of a patient in intensive care with a hospital-acquired pneumonia that did not respond to meropenem therapy. The strain is resistant to ceftazidime, imipenem and piperacillin-tazobactam and displays intermediate resistance to meropenem and aztreonam. The isolate was positive for the modified carbapenemase inhibition test and the carbapenem inactivation method by the Scottish AMR Satellite Reference Laboratory, Glasgow^21^. It was not found to possess any known carbapenemase enzymes at the Antimicrobial Resistance and Healthcare Associated Infections Reference Unit (AMRHAI), Public Health England, Colindale, and the antibiotic resistance profile was consistent with loss of the OprD porin and enhanced drug efflux. Finally, *P*. *aeruginosa* PA01 was provided by Dr. Olga Lomovskaya, Rempex Pharmaceuticals, USA. All strains were grown to stationary phase in Mueller–Hinton broth (MHB; Merck, Darmstadt, Germany) at 37 °C with shaking (at 200 rpm) overnight to prepare inocula for antibiotic efficacy testing *in vivo*.

### Antibiotics and *G*. *mellonella* larvae

All antibiotics were purchased from Sigma–Aldrich Ltd (Dorset, UK). Stock solutions of antibiotics were prepared in sterile deionized water. *G*. *mellonella* larvae were obtained from UK Waxworms Ltd. (Sheffield, UK).

### Antibiotic susceptibility testing

Minimum inhibitory concentrations (MICs) of antibiotics against each of the *P*. *aeruginosa* strains were determined in 96-well microplates as previously described^22^. Briefly, doubling dilutions of each antibiotic were prepared in MHB and subsequently inoculated with 1.0 × 10^6^ cfu/mL of *P*. *aeruginosa*. Microplates were incubated at 37 °C and the MIC was defined as the concentration(s) present in the first optically clear well after 24 h.

### *G*. *mellonella* infection model

*G*. *mellonella* at their final instar larval stage were kept at room temperature in darkness. Larvae weighing within the range of 250 to 350 mg were selected for each experiment to ensure consistency in subsequent drug administration and were used within 1 week of receipt.

Efficacy of antibiotics alone or in combination versus *G*. *mellonella* larvae infected with the *P*. *aeruginosa* strains was carried out exactly as described previously^22–24^. Briefly, groups of 15 larvae were infected with an inoculum of 2.5 × 10^3^ cfu/mL of *P*. *aeruginosa* cells (unless otherwise stated). Treatment with a single dose of each antibiotic alone, or dual combinations of these antibiotics, were administered 2 h post-infection. The experiments were repeated in duplicate using larvae from a different batch and the data from these replicate experiments were pooled to give *n* = 30. Survival data were plotted using the Kaplan–Meier method^25^ and comparisons made between groups using the log-rank test^26^. In all comparisons with the negative control it was the uninfected control (rather than the unmanipulated control) that was used. Holm’s correction was applied to account for multiple comparisons in all tests and *P* ≤ 0.05 was considered significant^27^.

### PBP detection and β-lactam titration

PBP detection and β-lactam titration were carried out as previously described with several minor alterations^28^. *P*. *aeruginosa* PAO1 cells from 1.5 ml of an overnight culture were harvested by centrifugation at 8,000 × *g* for 3 minutes at room temperature, washed in 1 mL of phosphate buffered saline (PBS), harvested, and resuspended in 80 μL of PBS containing 0.0001–1000 mg/L of aztreonam, ceftazidime or meropenem alone, or 0.0002–2000 mg/L of aztreonam + meropenem or ceftazidime + meropenem. A reference sample was resuspended in 80 μl of PBS alone. The cells were incubated at 37 °C for 40 mins prior to harvesting and washing in PBS, harvesting and resuspension in 50 μL of PBS containing 30 μg/mL of BOCILLIN™ FL penicillin, sodium salt (Boc-FL), a fluorescent analogue of penicillin V coupled with the fluorophore BODIPY FL, used to detect and label PBPs (Invitrogen, Eugene, Oregon). The cells were incubated at 37 °C for 30 mins, prior to harvesting and washing and final resuspension in 100 μL of PBS, and then sonicated (Hielscher Ultrasonic Processor (UPS200S)) for 4 × 10 second intervals (cycle = 0.5, amplitude = 40%) with 10 seconds cooling between each round. Following sonication, any remaining un-lysed cells were harvested (8,000 × *g* for 3 minutes at 4 °C) and the supernatant collected. The protein content was measured (NanoDrop 2000, Thermo Scientific) and all samples were adjusted to 8 mg/mL via dilution with PBS. Next, 51 μL of each protein sample was mixed with 17 μL of 4 × Laemmli sample buffer (Bio-Rad, Watford, UK) supplemented with β-mercaptoethanol (Sigma-Aldrich, Dorset, UK) as described in the manufacturer instructions, and de-natured at 95 °C for 5 mins before loading 20 μL onto a 10% Tris-HCl polyacrylamide gel (Criterion™, Bio-Rad, Watford, UK). Proteins were separated via gel-electrophoresis for 1 hr 45 min at 120 V. The gels were then rinsed repeatedly with deionised water and imaged (excitation at 473 nm with a 520 nm emission filter at 50-μm pixel resolution) using a Typhoon FLA 7000 scanner (GE Healthcare Life Sciences, Little Chalfont, Bucks., UK).

### Image J analysis and calculation of half maximal inhibitory concentration (IC_50_)

Antibiotic PBP affinity profiles and IC_50_ values were determined as described previously^28^. Briefly, gel images were imported into Image J software (https://imagej.nih.gov/ij/download.html) and the brightness and contrast levels were adjusted to optimise the signal to noise ratio. The background was subtracted (rolling ball radius: 60 pixels), and dark noise outliers were removed (radius: 3 pixels, threshold: 50) to reduce the appearance of blemishes on the Typhoon scanner stage. The density of Boc-FL labelling in each protein band was quantified relative to the no-antibiotic reference sample using the Analyse Gels function (http://lukemiller.org/index.php/2010/11/analyzing-gels-and-western-blots-with-image-j/). Relative Boc-FL labelling values from two independent experiments were averaged and GraphPad Prism (GraphPad Software, La Jolla, CA) was used to create graphs showing relative % Boc-FL labelling versus β-lactam concentration. The binding affinity of each PBP for each antibiotic was determined by fitting a log_10_(inhibitor) vs. response – variable slope (four-parameter) curve to the % inhibition data from two independent experiments. IC_50_ values were determined using a 4-parameter logistic regression equation using GraFit 5.0 (Erithacus Software, East Grinstead, UK).

### Determination of circulating haemocyte numbers

Groups of 15 larvae per condition were injected with 10 µL of PBS followed 2 h later with either PBS, aztreonam (50 mg/kg), ceftazidime (5 mg/kg) or meropenem (2.5 mg/kg) or aztreonam + meropenem (50 + 2.5 mg/kg) or ceftazidime + meropenem (5 + 2.5 mg/kg) to assess the effects of these antibiotic treatments on circulating haemocyte numbers. Unmanipulated controls were also included. Three larvae were randomly selected and injected with 75 µL *Galleria* saline^29^ at 5, 20.5 and 24 hours post-infection and haemolymph was bled into individual sterile reaction tubes and 10 µL was loaded onto an improved Neubauer haemocytometer and the haemocytes counted with duplicates for each sample. This experiment was performed in triplicate to give *n* = 9 for each experimental condition.

## Results

### Treatment of *P*. *aeruginosa* infected *G*. *mellonella* with dual combinations of a range of β-lactam antibiotics results in enhanced efficacy compared to monotherapy

A group of eight β-lactam antibiotics [meropenem (MEM), ampicillin (AMP), piperacillin (PIP), aztreonam (ATM), cefuroxime (CXM), cefotaxime (CTX), cefadroxil (CFR) and ceftazidime (CAZ)] that each represent a common class of this group of drugs were selected for screening of *in vivo* efficacy in dual combinations (a total of 28 combinations). *G*. *mellonella* larvae were infected with *P*. *aeruginosa* NCTC13437 because it is a characterised MDR strain that has been used successfully to infect *G*. *mellonella* in previous studies^22,24^. Initial pilot experiments determined the efficacy of monotherapy (a single dose, administered 2 h post-infection (p.i)) with each of the β-lactam antibiotics listed above on *G*. *mellonella* larvae infected with a lethal dose (2.5 × 10^3^ cells/mL) of *P*. *aeruginosa* NCTC13437. Four of the β-lactams administered (AMP, CXM, CTX and CFR) resulted in minimal therapeutic benefit to infected larvae at the highest dose tested (100 mg/kg). This result is consistent with the fact that these β-lactams have minimal anti-pseudomonal activity. In contrast, PIP, CAZ, ATM and MEM each resulted in enhanced survival of infected larvae in a dose dependent manner (data not shown). These initial experiments allowed the selection of doses of each individual antibiotic for subsequent study of the effect of dual combinations - doses of each constituent antibiotic that had minimal therapeutic benefit as monotherapies were selected. This approach allowed optimal identification of combinations that offer enhanced efficacy compared to the constituent monotherapies.

The effect of the 28 possible dual β-lactam combinations on survival of *G*. *mellonella* larvae infected with a lethal dose of *P*. *aeruginosa* NCTC13437 (2.5 × 10^3^ cells/mL) is shown in Table 1. Many of the dual combinations showed enhanced survival compared to either monotherapy or treatment with PBS. Five of the dual combinations (MEM + PIP; MEM + ATM; MEM + CAZ; PIP + ATM and ATM + CTX) resulted in greater than 70% survival after 96 h p.i. Markedly, all of these five most potent combination treatments contained either MEM or ATM, or both.

**Table 1:** Initial screen of the efficacy of twenty-eight dual β-lactam combination treatments against *G*. *mellonella* larvae infected with a lethal dose of *P*. *aeruginosa* NCTC13437.

Therapy	Antibiotic(s) or PBS control	Dose (mg/kg)	% survival *in vivo* 96 h p.i
Sham treatment	PBS	10 μL PBS	0
Monotherapy	MEM	2.5	3
	AMP	100	0
	PIP	100	30
	ATM	50	23
	CXM	100	7
	CTX	100	27
	CFR	100	0
	CAZ	5	10
Dual combination therapy	MEM + AMP	2.5 + 100	33
	MEM + PIP	2.5 + 100	73*
	MEM + ATM	2.5 + 50	87*
	MEM + CXM	2.5 + 100	60*
	MEM + CTX	2.5 + 100	40
	MEM + CFR	2.5 + 100	33
	MEM + CAZ	2.5 + 5	80*
	AMP + PIP	100 + 100	40
	AMP + ATM	100 + 50	47
	AMP + CXM	100 + 100	0
	AMP + CTX	100 + 100	7
	AMP + CFR	100 + 100	0
	AMP + CAZ	100 + 5	0
	PIP + ATM	100 + 50	73*
	PIP + CXM	100 + 100	20
	PIP + CTX	100 + 100	53
	PIP + CFR	100 + 100	7
	PIP + CAZ	100 + 5	13
	ATM + CXM	50 + 100	53
	ATM + CTX	50 + 100	80*
	ATM + CFR	50 + 100	47
	ATM + CAZ	50 + 5	60
	CXM + CTX	100 + 100	27
	CXM + CFR	100 + 100	0
	CXM + CAZ	100 + 5	7
	CTX + CFR	100 + 100	7
	CTX + CAZ	100 + 5	40
	CFR + CAZ	100 + 5	0

One dose of each monotherapy or dual combination was administered 2 h p.i. and survival measured 96 h p.i. *Indicates significantly enhanced survival compared to both monotherapies (*p* < 0.05, log rank test with Holm correction for multiple comparisons). n = 15.

**Figure 1:**
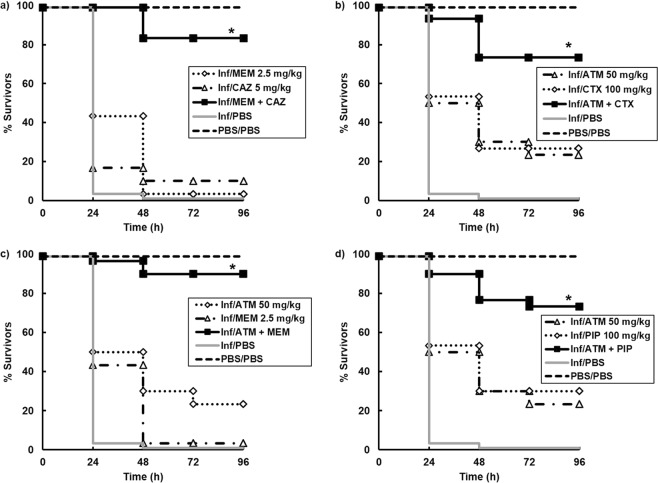
Effect of treatment with β-lactam monotherapies or dual combinations on survival of *G*. *mellonella* larvae infected with 2.5 × 10^3^ cfu/mL *P*. *aeruginosa* NCTC13437 and treated with PBS or: (**a**) MEM (2.5 mg/kg), CAZ (5 mg/kg) or MEM + CAZ (2.5 + 5 mg/kg); (**b**) ATM (50 mg/kg), CTX (100 mg/kg) or ATM + CTX (50 + 100 mg/kg); (**c**) ATM (50 mg/kg), MEM (2.5 mg/kg) or ATM + MEM (50 + 2.5 mg/kg); (**d**) ATM (50 mg/kg), PIP (100 mg/kg) or ATM + PIP (50 + 100 mg/kg). A single dose of the antibiotic treatments was administered 2 h p.i. The uninfected group represents larvae sham-infected with sterile PBS and treated with sterile PBS. *Indicates significantly enhanced survival compared to both monotherapies (*p* < 0.05, log rank test with Holm correction for multiple comparisons); *n* = 30 (pooled from duplicate experiments).

**Figure 2:**
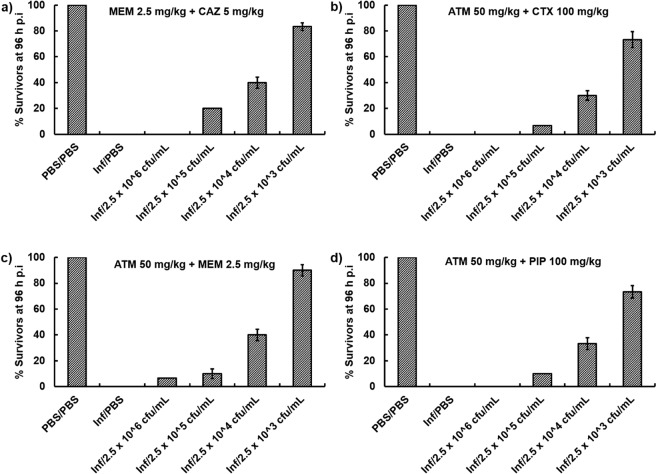
Survival of *G*. *mellonella* larvae 96 h p.i with increasing inoculum sizes (2.5 × 10^3^, 10^4^, 10^5^ or 10^6^ cfu/mL) of *P*. *aeruginosa* NCTC13437 and treated with PBS or dual β-lactam combinations of: (**a**) MEM + CAZ (2.5 + 5 mg/kg); (**b**) ATM + CTX (50 + 100 mg/kg); (**c**) ATM + MEM (50 + 2.5 mg/kg); (**d**) ATM + PIP (50 + 100 mg/kg). A single dose of the antibiotic treatments was administered 2 h p.i. The uninfected group represents larvae sham-infected with sterile PBS and treated with sterile PBS. Error bars represent the mean ± SEM of replicate experiments.

Following this initial screen, four of the best combinations were studied in greater detail and confirmed significantly enhanced efficacy of MEM + CAZ, ATM + CTX, ATM + MEM and ATM + PIP compared to sham treatment with either PBS or each constituent monotherapy (Fig. 1). To test the potency of the above combinations, the therapeutic benefit of each combination treatment was determined versus *G*. *mellonella* larvae infected with increasing inoculum sizes of *P*. *aeruginosa* NCTC13437 (Fig. 2). As shown before, each of the four combination treatments significantly enhanced survival of larvae infected with 2.5 × 10^3^ c.f.u/mL 96 h p.i. Increasing the number of infecting bacteria 10-fold (2.5 × 10^4^ c.f.u/mL), 100-fold (2.5 × 10^5^ c.f.u/mL) or 1000-fold (2.5 × 10^6^ c.f.u/mL) resulted in an inoculum-dependent reduction in survival for all combinations tested. Notably, all four combinations still had some therapeutic benefit 96 h p.i with 2.5 × 10^5^ c.f.u/mL and treatment with ATM + MEM resulting in some survival after infection with the highest inoculum tested - 2.5 × 10^6^ c.f.u/mL (Fig. 2).

In summary, *G*. *mellonella* larvae infected with an MDR strain of *P*. *aeruginosa* were successfully treated with dual combinations of β-lactam antibiotics.

### Dual combinations of β-lactam antibiotics also result in enhanced survival of *G*. *mellonella* larvae infected with an alternative antibiotic resistant, clinical isolate of *P*. *aeruginosa*

**Figure 3:**
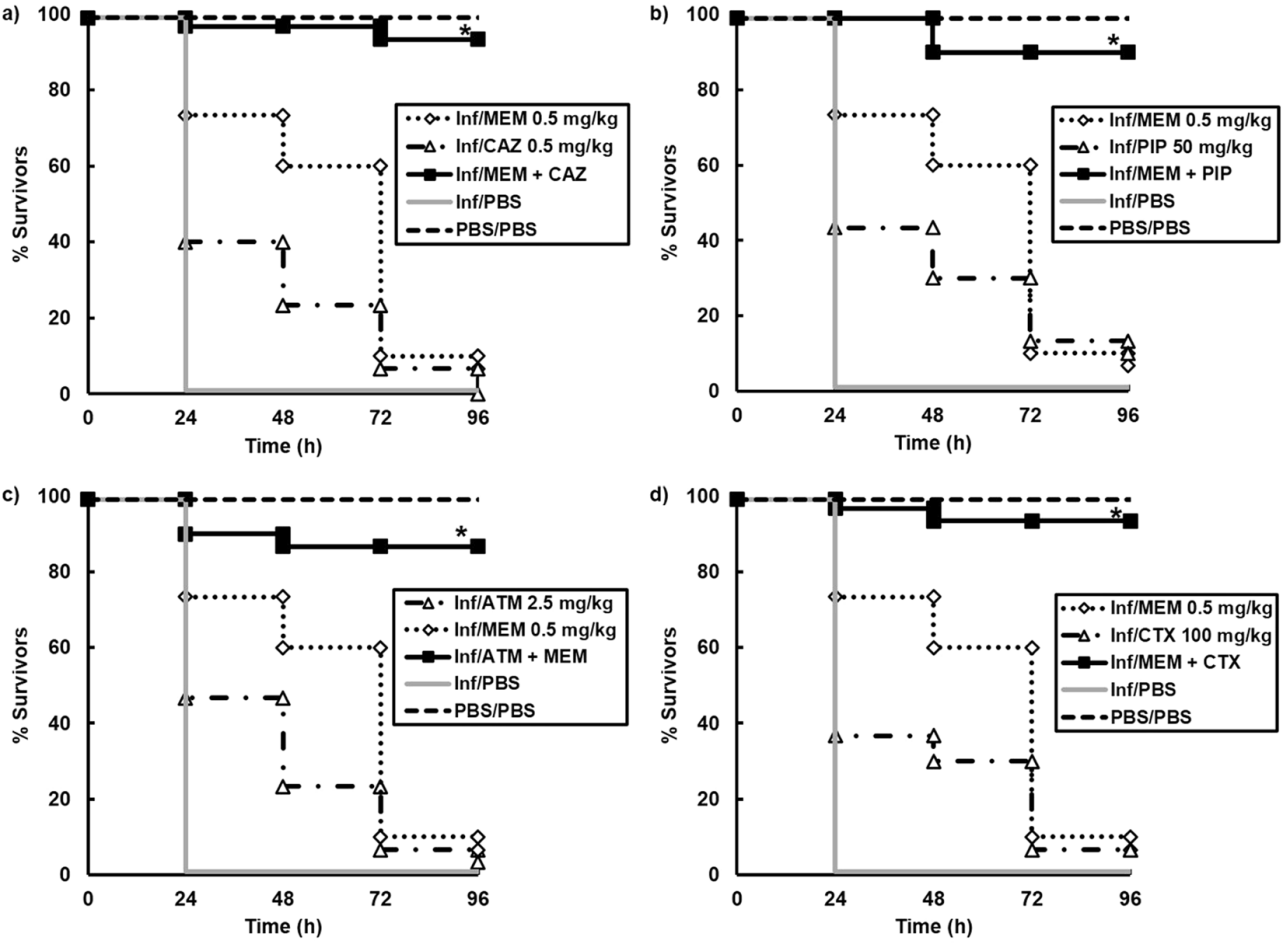
Effect of treatment with β-lactam monotherapies or dual combinations on survival of *G*. *mellonella* larvae infected with 2.5 × 10^3^ cfu/mL of a carbapenem-resistant clinical isolate of *P*. *aeruginosa* and treated with PBS or: (**a**) MEM (0.5 mg/kg), CAZ (0.5 mg/kg) or MEM + CAZ (0.5 + 0.5 mg/kg); (**b**) MEM (0.5 mg/kg), PIP (50 mg/kg) or MEM + PIP (0.5 + 50 mg/kg); (**c**) ATM (2.5 mg/kg), MEM (0.5 mg/kg) or ATM + MEM (2.5 + 0.5 mg/kg); (**d**) MEM (0.5 mg/kg), CTX (100 mg/kg) or MEM + CTX (0.5 + 100 mg/kg). A single dose of the antibiotic treatments was administered 2 h p.i. The uninfected group represents larvae sham-infected with sterile PBS and treated with sterile PBS. *Indicates significantly enhanced survival compared to both monotherapies (*p* < 0.05, log rank test with Holm correction for multiple comparisons); *n* = 30 (pooled from duplicate experiments).

To determine if the same potent β-lactam combinations resulted in enhanced efficacy versus an alternative antibiotic-resistant *P*. *aeruginosa* strain, the therapeutic effect of the combinations was tested on larvae infected with a carbapenem-resistant clinical isolate of *P*. *aeruginosa*. As before, initial experiments measured the effect of β-lactam monotherapies to identify doses of each drug that resulted in minimal therapeutic benefit to infected larvae that could then be administered in the combination studies (data not shown). The same dual combinations of β-lactams administered to *P*. *aeruginosa* NCTC13437 (Table 1) were administered to larvae infected with the clinical isolate. Once again, many combinations resulted in enhanced efficacy compared to their constituent monotherapies and the four best combination treatments (MEM + CAZ, ATM + MEM, MEM + PIP and MEM + CTX) are shown in Fig. 3. As before, these four most potent combination treatments contained either MEM or ATM, or both. Notably, two combinations showed potent, enhanced efficacy versus both strains of *P*. *aeruginosa* used in this study - MEM + CAZ and ATM + MEM and subsequent studies focused on these two combinations.

To conclude, the significantly enhanced efficacy of dual combinations of β-lactam antibiotics observed previously was replicated against an unrelated, antibiotic-resistant clinical isolate of *P*. *aeruginosa*.

### The enhanced efficacy of the MEM + CAZ and ATM + MEM combinations cannot be explained by increased inhibition of a wider range of *P*. *aeruginosa* PBPs

A primary hypothesis explaining why β-lactam combinations can offer enhanced efficacy is the broadened spectrum of PBPs that could be inhibited by administering multiple drugs with different PBP affinities [reviewed in^19^]. Thus, the effect of the most potent combinations on inhibition of *P*. *aeruginosa* PBPs was compared with the effect of the constituent monotherapies. For this work, *P*. *aeruginosa* PA01 was used rather than the antibiotic-resistant strains employed previously because the presence of VEB-1 and VIM-10 in NCTC13437, could interfere with the PBP-labelling assay that uses Bocillin-FL (Boc-FL), a fluorescent analogue of penicillin V, to measure antibiotic-dependent inhibition of PBPs.

**Figure 4:**
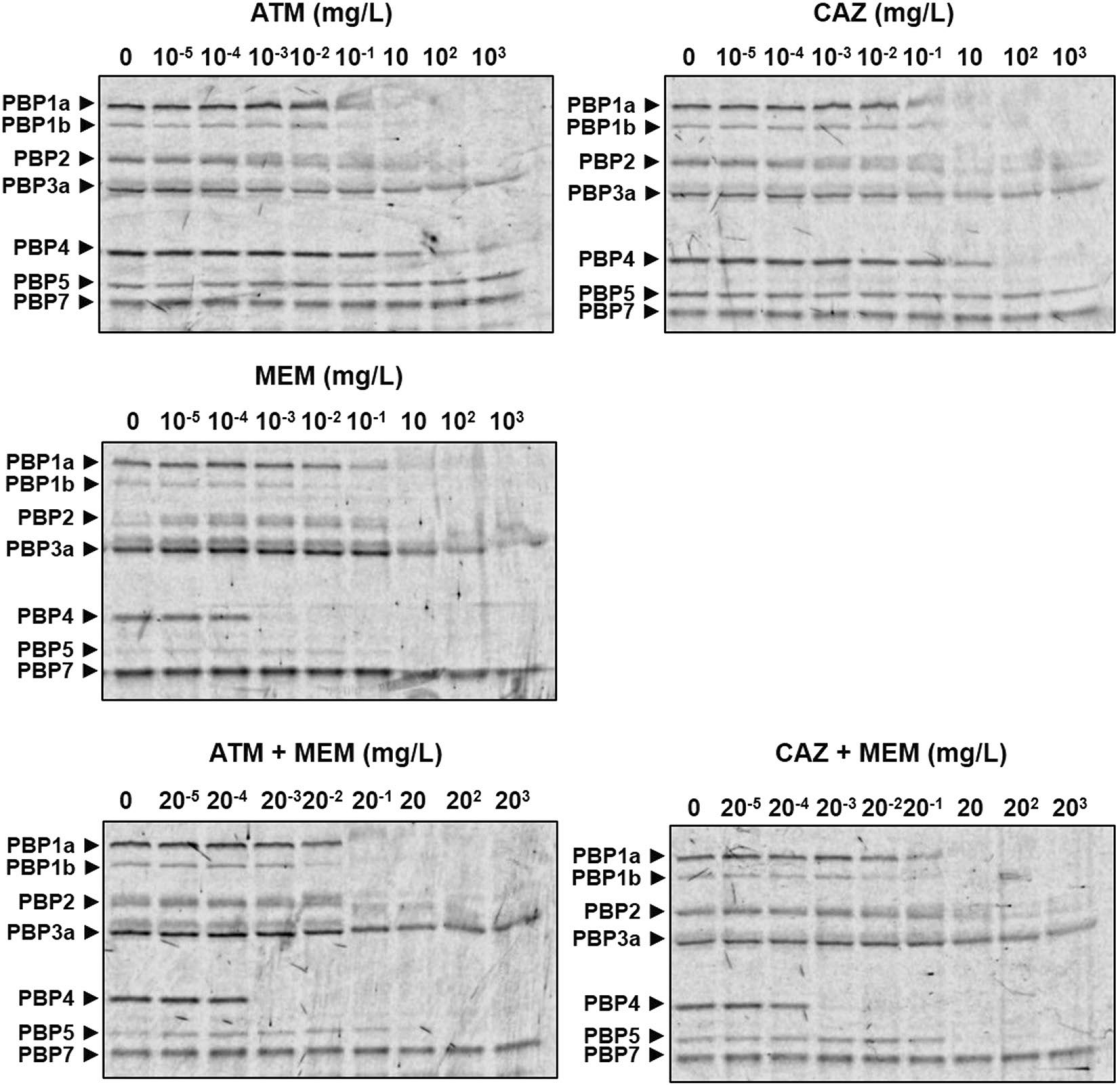
Titration of *P*. *aeruginosa* PA01 PBPs by ATM, CAZ or MEM, or ATM + MEM, or CAZ + MEM. Live cells were treated with 10-fold increasing concentrations of each of the β-lactam treatments listed above prior to labelling of PBPs with a fluorescent analogue of penicillin V (Boc-FL). Cells were sonicated and the protein content in each sample equalised to 8 mg/mL. PBPs were separated via SDS-PAGE on 10% Tris-HCl polyacrylamide gels and visualised using a fluorescent scanner (excitation at 473 nm with a 520 nm emission filter). Gel images shown are representative images from duplicate experiments.

Boc-FL has been used to study the PBPs of a range of bacteria, including *P*. *aeruginosa*^30–33^. Live *P*. *aeruginosa* PA01 cells were incubated with increasing concentrations of CAZ, MEM or ATM and the dual combinations of MEM + CAZ or ATM + MEM. After incubation, PBPs that had not been bound by the single antibiotics or combinations were labelled using Boc-FL. The PBPs were separated by SDS-PAGE and the affinity of the β-lactams or the combinations for each PBP was quantified by measuring Boc-FL labelling relative to an unexposed control. Experiments were run in duplicate and an example gel image for each of the drug treatments and their effect on PBP labelling by Boc-FL is shown in Fig. 4. Seven gel bands corresponding to PBP1a, PBP1b, PBP2, PBP3a, PBP4, PBP5 and PBP7 were identified. Identification of the *P*. *aeruginosa* PA01 PBPs was based on molecular weight and comparison of the electrophoretic pattern with previously published studies^30–33^.

**Figure 5:**
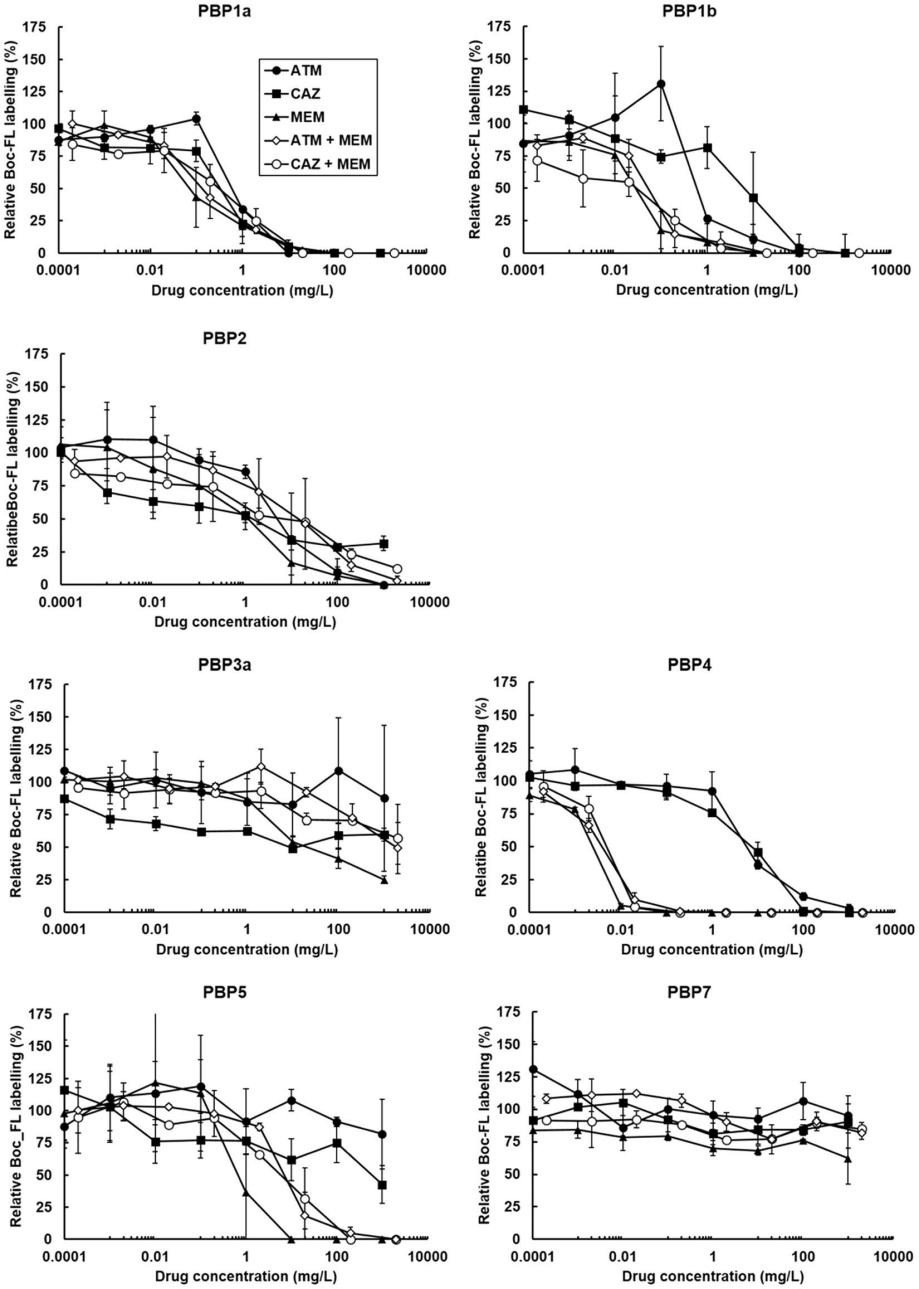
Quantitation of Boc-FL labelling of each *P*. *aeruginosa* PA01 PBP gel band in the presence of increasing concentrations of either ATM, CAZ or MEM, or the dual combinations of ATM + MEM or CAZ + MEM. The density of Boc-FL labelling of each PBP gel band was quantified relative to the no-antibiotic exposure reference sample and expressed as %. Relative Boc-FL labelling values from two independent experiments were averaged and error bars represent the mean ± SEM.

Dose-dependent inhibition of Boc-FL labelling of PBPs upon exposure to increasing concentrations of the different β-lactam treatments can be observed on the gels and clear differences in PBP affinities between the different treatments can be seen. For example, there is visible loss of PBP4 Boc-Fl labelling in cells exposed to 10^−3^ mg/L of MEM that is not seen with either ATM or CAZ at the same concentration (Fig. 4). Quantitative analysis of Boc-FL labelling of PBPs was performed using densitometry of the gel bands representing each of the *P*. *aeruginosa* PBPs. The density of the PBP bands from control cells not exposed to any β-lactams were set at 100% and the densities of the PBP bands on the gel after exposure to increasing concentrations of the single β-lactams, or the two dual combinations, were expressed as a percentage of the control. Thus, decreasing Boc-FL labelling of PBPs results in a reduction in gel band densities and indicates increasing inhibition of PBPs by the β-lactam regimen being tested. Figure 5 shows the relative % of Boc-FL labelling against β-lactam concentration for each of the PBPs. Exposure to increasing concentrations of ATM caused an initial increase in Boc-FL labelling of PBP1a and PBP1b relative to the control before inhibition occurred. This effect has been documented before when ATM was shown to cause an increase in labelling of these PBPs in *Escherichia coli*^28^. No increase in protein levels were detected and the increase in labelling remains unexplained.

Differences in the affinities of the β-lactam regimens for PBPs can be observed. For example, MEM is the most potent inhibitor of PBP1a with >50% inhibition of Boc-FL labelling at 0.1 mg/L compared to approximately 25% inhibition for CAZ, and no apparent inhibition for ATM, at the same concentration. Similarly, at 0.1 mg/L MEM is clearly a more potent inhibitor of PBP1b than either ATM or CAZ. With PBP2 and 3a obvious differences between the different β-lactam treatments inhibition profiles were indiscernible. The most significant difference in affinity between the β-lactams was observed for PBP4. At 1 mg/L, MEM reduced Boc-Fl binding by 100% compared to an approximate 25% and 10% reduction by CAZ and ATM respectively. MEM also appeared to be a more potent inhibitor of PBP5 than either CAZ or ATM. None of the β-lactam treatments inhibited PBP7 in a dose-dependent manner and >50% reduction in Boc-Fl labelling was not observed at any concentration tested. Notably, the inhibition profiles of the dual combinations ATM + MEM and CAZ + MEM appeared to closely match that of MEM alone but without any obvious enhancement of affinity for any of the PBPs. In fact, for PBP5, the combinations did not inhibit as potently as MEM alone despite having a more potent inhibitory effect than either ATM or CAZ.

To describe the PBP affinity profile of each of the β-lactam treatments, half maximal inhibitory concentration (IC_50_) values were determined and are shown in Table 2. Dose-response curves were fitted to the data describing the inhibition of each PBP by each of the β-lactam treatments tested and IC_50_ values calculated. The IC_50_ values provide insight into the preferential PBP targets for each of the treatments and relating the IC_50_ concentration with the MIC of *P*. *aeruginosa* PA01 also indicates if the PBP inhibition is likely to contribute to the antibacterial effect of the drug. The MIC values (mg/L) for *P*. *aeruginosa* PA01 were: ATM (1–2); CAZ (0.5–1) and MEM (1). Thus, ATM was selective for PBP1a and PBP1b and the IC_50_ values for both PBPs were less than the MIC for this drug. CAZ was selective for PBP1a and PBP2 and IC_50_ values for both PBPs were lower than the MIC, particularly that for PBP2. MEM was clearly the most potent β-lactam tested in terms of PBP affinity, showing selectivity for PBP1b and PBP4 but the IC_50_ values for PBP1a, 1b, 2, 4 and 5 were all lower than the MIC. Remarkably, none of the PBP IC_50_ values for the dual β-lactam combinations, both of which contained MEM, were lower than those obtained for MEM alone. With the exception of PBP4, the IC_50_ values were all higher than those calculated for MEM. From the dose-response curves, the % inhibition of each PBP by each individual antibiotic at their MIC was calculated and is shown in Table 3. This data supports the previous observations and implies that inhibition of PBPs by a combination of MEM with ATM or CAZ is not likely to be greater than the degree of inhibition induced upon exposure to MEM alone.

**Table 2:** IC_50_ values (± the standard error determined in GraFit 5.0) for *P*. *aeruginosa* PAO1 PBPs for the single antibiotic treatments (ATM, CAZ or MEM) and the dual combination treatments (MEM + ATM and MEM + CAZ).

Beta-lactam treatment	IC_50_ (mg/L)						
	PBP1a	PBP1b	PBP2	PBP3a	PBP4	PBP5	PBP7
ATM	0.904 ± 0.283	0.816 ± 0.543	4.56 ± 3.746	>1000	5.449 ± 2.035	>1000	>1000
CAZ	0.469 ± 0.159	4.48 ± 4.33	1.329 ± 1.134	>1000	8.022 ± 2.19	>1000	>1000
MEM	0.105 ± 0.053	0.037 ± 0.009	0.744 ± 1.251	6.034 ± 4.878	0.003 ± 0.0002	0.3697 ± 0.271	>1000
ATM + MEM	0.158 ± 0.068	0.067 ± 0.022	15.884 ± 19.289	>1000	0.004 ± 0.0011	5.498 ± 4.649	>1000
CAZ + MEM	0.631 ± 0.326	0.082 ± 0.042	17.987 ± 26.859	>1000	0.004 ± 0.0005	6.724 ± 2.874	>1000

Underlining shows lowest IC_50_ for each β-lactam treatment − indicating selectivity.

**Table 3:** Degree of inhibition of *P*. *aeruginosa* PAO1 PBPs at the approximate MIC (1 mg/L) of each of the individual antibiotics.

Beta-lactam treatment	% Inhibition of Boc-FL labelling at MIC (1 mg/L)						
	PBP1a	PBP1b	PBP2	PBP3a	PBP4	PBP5	PBP7
ATM	65.28	69.07	18.13	0	9.97	0	0
CAZ	76.49	28.65	50.1	0	21.64	0	0
MEM	86.03	97.5	52.59	14.76	100	54.73	0

In summary, the enhanced efficacy of the dual β-lactam combinations ATM + MEM and CAZ + MEM against *P*. *aeruginosa in vivo* is not due to more potent inhibition of PBPs or inhibition of a broader range of PBPs.

### Exposure of uninfected *G*. *mellonella* larvae to the dual β-lactam combinations ATM + MEM and CAZ + MEM stimulates an increase in circulating haemocyte numbers

Because the enhanced efficacy of the dual β-lactam combinations cannot be explained by increased inhibition of PBPs, alternative mechanisms were considered. In a previous study using *G*. *mellonella*, administration of antibiotics alone to uninfected larvae induced a significant increase in the number of circulating haemocytes compared to larvae injected with PBS^34^. Thus, perhaps the enhanced efficacy of the dual β-lactam combinations could be explained by antibiotic-mediated stimulation of innate immunity in a similar fashion.

**Figure 6:**
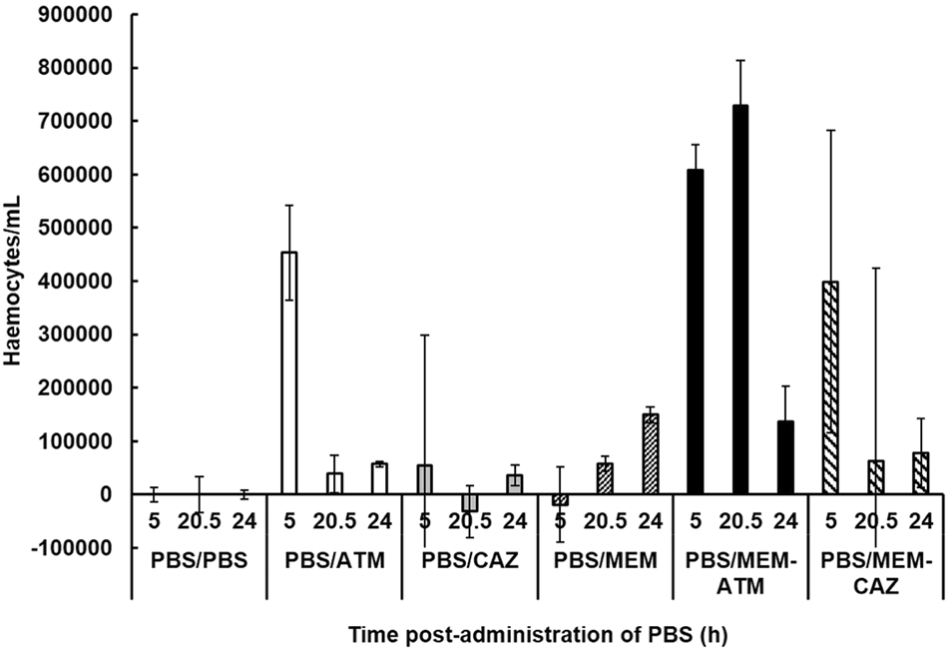
Effect of exposure to β-lactams on overall numbers of circulating *G*. *mellonella* haemocytes. Larvae were sham-infected with PBS and then 2 h p.i administered either PBS, ATM (50 mg/kg), CAZ (5 mg/kg) or MEM (2.5 mg/kg), or combinations of ATM + MEM or CAZ + MEM at the same doses as the single drugs. Numbers of haemocytes in the larval haemolymph were then counted at 5, 20.5 and 24 h p.i. Haemocyte numbers are presented relative to larvae sham-infected with PBS and subsequently treated with PBS. Data is from 9 larvae per time-point showing mean ± SEM.

The effect of administration of ATM, CAZ and MEM alone and ATM + MEM or CAZ + MEM to uninfected *G*. *mellonella* larvae on the numbers of circulating haemocytes was measured using microscopy (Fig. 6). Larvae were mock-infected with sterile PBS then injected 2 h later with PBS, ATM (50 mg/kg), CAZ (5 mg/kg) or MEM (2.5 mg/kg), or combinations of ATM + MEM or CAZ + MEM at the same doses as the single drugs. Relative to PBS treatment, exposure to ATM resulted in an initial, transient increase in haemocyte numbers after 5 h. Exposure to MEM alone also resulted in an increase but less than ATM with the maximum increase occurring after 24 h. In contrast, CAZ alone resulted in only a minor increase in haemocyte number after 24 h. Notably, exposure to either of the dual β-lactam combinations resulted in larger, more significant increases in circulating haemocytes than the individual drugs (Fig. 6).

In summary, antibiotic-mediated stimulation of innate immunity in the form of increased haemocyte numbers could contribute to the enhanced efficacy of the β-lactam combinations *in vivo*.

## Discussion

Effective β-lactam combinations that target *P*. *aeruginosa* have been identified in previous studies but were never developed further for clinical administration principally due to lack of need (reviewed in^19^). However, the rise in problematic MDR Gram-negative infections with few treatment options means that β -lactam combinations could be a potential solution. This study presents evidence that dual combinations of β-lactams offer enhanced efficacy against infections caused by MDR *P*. *aeruginosa* in an invertebrate model of systemic infection. The two most potent dual combinations identified in this study (meropenem with aztreonam or ceftazidime) have not been reported previously. The presence of a carbapenem (meropenem) in these effective combinations is notable because much of the past research on β-lactam combinations was carried out in the 1980’s and data is lacking on the use of carbapenems in combinations versus *P*. *aeruginosa*, particularly *in vivo*. Furthermore, the majority of the previous studies on the efficacy of β-lactam combinations were not carried out on MDR strains of *P*. *aeruginosa*^19^. Thus, the effective combinations reported here could represent real options for therapeutic intervention against resistant *P*. *aeruginosa* and merit further investigation in mammalian infection models and ultimately patients. This conclusion is supported by a number of studies that have highlighted successful application of dual β-lactam combinations to treat patients infected with MDR, and carbapenemase carrying, strains of *Klebsiella pneumoniae*^35–38^.

Many of the potent β-lactam combinations that were identified in this study included the synthetic, monocyclic β-lactam, aztreonam. This observation is supported by many *in vitro* studies that report significant synergy versus *P*. *aeruginosa* between aztreonam and third or fourth generation cephalosporins, such as ceftazidime and cefepime^19,39^. The inclusion of aztreonam in dual combinations versus MDR Gram-negative bacteria that possess carbapenemases is logical because the drug is not hydrolysed by MBLs. However, aztreonam is susceptible to hydrolysis by most serine ESBLs and because many MDR pathogens carrying an MBL also possess ESBLs, aztreonam will need to be administered in combination with the novel β-lactamase inhibitor avibactam (reviewed in^40^). Unfortunately, avibactam is only a weak inhibitor of Ambler class B MBLs such as VIM-10 that is carried by *P*. *aeruginosa* NCTC 13437 used in this study and is thus unlikely to be part of an effective therapy^41^.

Definitive studies on the actual mechanism underpinning β-lactam synergy are lacking. However, the principal hypothesis is that a broader spectrum of PBPs can be inhibited by combining β-lactams with different PBP affinity profiles. While β-lactams confer their antibiotic activity through PBP inhibition, individual antibiotics differ in their affinities for each of the bacterial PBPs. Therefore, a combination treatment with two β-lactams each with differing PBP binding profiles could provide broader PBP inhibition and thus collectively enhance their inhibitory effect and efficacy *in vivo*^19^. Adding to this, an alternative ‘shielding’ hypothesis potentially explains why dual β-lactams are more effective against resistant strains possessing ESBLs or MBLs - one of the component β-lactams could bind preferentially, or with higher affinity, to the β-lactamase enzyme, thus sequestering the hydrolytic capacity of the enzyme, and allowing the other component β-lactam to bind to its target PBP(s) more effectively. For example, Lister *et al*.^42^. revealed that aztreonam enhanced the activity of cefepime versus *P*. *aeruginosa* strains expressing chromosomal AmpC β-lactamases. A possible explanation for this was the high affinity aztreonam has for the active site of some β-lactamases, such as AmpC enzymes, meaning that it could also act as an inhibitor thus permitting cefepime to act unhindered^43^.

In this work, a novel method first used to study *Escherichia coli* PBPs was applied to *P*. *aeruginosa*^28^. This method uses Boc-FL to label PBPs in living cells rather than purified membranes with the advantage of avoiding any negative effects membrane preparation could have on PBP activity^44^. Using this method, all *P*. *aeruginosa* PBPs were identified with the exception of PBP3. PBP3 was not seen because stationary-phase cells were used in these experiments. PBP3 is expressed during the exponential-phase of growth and is down-regulated upon entry to stationary phase when cells switch to PBP3a expression^45^. Stationary phase cells were used to be consistent with the earlier experiments measuring efficacy of the various β-lactam treatments on *G*. *mellonella* larvae that were infected with stationary phase cells. PBP3 is essential for growth of *P*. *aeruginosa*, while the other PBPs can be individually deleted^46^. Many antipseudomonal β-lactams have been reported to preferentially bind to PBP3 including the drugs used in this study – aztreonam, ceftazidime and meropenem^30^. Unfortunately, because PBP3 was not detected here, any differences between the inhibition of PBP3 by the individual β-lactams and the dual combinations were not compared. However, if the previous studies are correct, and aztreonam, ceftazidime and meropenem all have high affinity for PBP3, it could be argued that combinations of these drugs would be unlikely to result in further significant inhibition that could explain the enhanced efficacy of the combinations observed *in vivo*.

PBP1a and PBP2 are thought to be relevant β-lactam targets as simultaneous mutation of their transpeptidase domain results in cells that show reduced fitness^46^. The low molecular weight (LMW) PBPs are not considered to be important targets of the β-lactams since simultaneous deletion of PBP4, PBP5 and PBP7 has no significant effect on growth rate or cell morphology^47^.

The calculated PBP affinities of the β-lactams used in this work with those reported previously were generally consistent but with some notable exceptions^19^. For example, ceftazidime was observed to inhibit PBP2 unlike previous studies^30,31^. Also, meropenem inhibited PBP5, and particularly PBP4, with high affinity and was also a more potent inhibitor of PBP1b than reported previously^19,30^. These discrepancies could be due to the use of different PBP labelling methods between this and previous studies as discussed above. Meropenem was found to inhibit multiple PBPs unlike aztreonam or ceftazidime. In addition, comparison of PBP inhibition at the MIC of all three drugs revealed that meropenem was also a more potent inhibitor of the PBPs that were preferentially targeted by either aztreonam or ceftazidime. This broad-spectrum of coverage of PBPs could explain why meropenem is the more potent antibiotic than the other drugs investigated when employed as a monotherapy.

The dual combinations of meropenem with either aztreonam or ceftazidime did not decrease the IC_50_ values below those that were calculated for meropenem alone. In fact, the combinations had increased IC_50_ values for all the PBPs compared with meropenem. Similarly, neither of the combinations resulted in broader coverage of overall PBPs inhibited compared to meropenem. Thus, the hypothesis that a broader spectrum of PBPs are inhibited by combining β-lactams with different PBP affinity profiles can be discounted as an explanation for the enhanced efficacy of these particular β-lactam combinations *in vivo*. Perhaps the ‘shielding’ hypothesis discussed previously offers the more likely explanation why PBP-inhibiting drug combinations show enhanced efficacy *in vivo* against β-lactamase carrying MDR strains of *P*. *aeruginosa* and additional studies will be required to test this hypothesis.

A further possible mechanism that could partly explain the enhanced efficacy of the β-lactam combinations *in vivo* is the increase in haemocyte numbers that was induced in response to exposure to the antibiotic combinations compared to the monotherapies. Notably, a direct correlation between increased numbers of circulating haemocytes and enhanced survival of *G*. *mellonella* larvae infected with fungal pathogens has been observed^48^. The immunomodulatory effects of antibiotics on human phagocytes can include both enhancement and suppression of phagocytosis (reviewed in^49^). β-lactams were previously shown to have pro-inflammatory properties by upregulating interleukin expression in murine macrophages^50^ and by stimulating macrophage phagocytosis^51^. Supporting the antibiotic-mediated increase in circulating haemocytes reported here, a similar immune-priming effect upon exposure to different antimicrobials has been shown in *G*. *mellonella* larvae previously^34,52,53^.

Irrespective of the precise inhibitory action, it is clear that combinations of PBP-inhibiting drugs show great promise in overcoming MDR *P*. *aeruginosa* and are worthy of additional study and development. Before dual β-lactam therapy can be optimised for MDR *P*. *aeruginosa* infections, it will be important to undertake additional studies to determine the contributing mode(s) of action in order to maximise bactericidal activity and prevent resistance development.

## Supplementary information

Supplementary Information

## Data Availability

The datasets generated during and/or analysed during the current study are available from the corresponding author on reasonable request.
