# Supplementary material for: Dual β-lactam combination therapy for multi-drug resistant Pseudomonas aeruginosa infection: enhanced efficacy in vivo and comparison with monotherapies of penicillin-binding protein inhibition
Source: Sci Rep. 2019 Jun 24;9:9098. doi: 10.1038/s41598-019-45550-z (PMC6591303; doi:10.1038/s41598-019-45550-z)

Dual β-lactam combination therapy for multi-drug resistant *Pseudomonas aeruginosa* infection: enhanced efficacy *in vivo* and comparison with monotherapies of penicillin-binding protein inhibition.

[1, 3]Thanyaluck Siriyong, [1, 2]Rachael M Murray, [1]Lucy E Bidgood, [1]Simon A Young, [1]Florence Wright, [4]Benjamin J Parcell, [5]Supayang Piyawan Voravuthikunchai, and [1]Peter J Coote*

Original gel scans imaged after excitation at 473 nm with a 520 nm emission filter at 50-μm pixel resolution using a Typhoon FLA 7000 scanner (GE Healthcare Life Sciences, Little Chalfont, Bucks., UK).

<u>Aztreonam titration</u>

Replicate 1 – original gel image

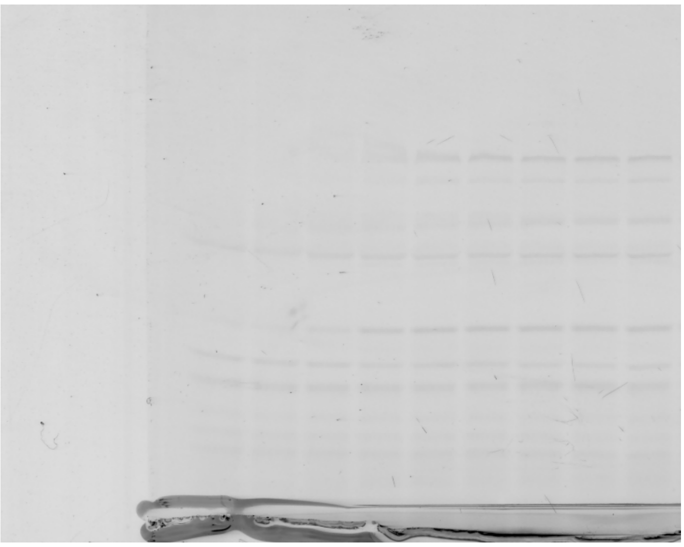

Replicate 1 - Adjusted image used for calculation of PBP inhibition

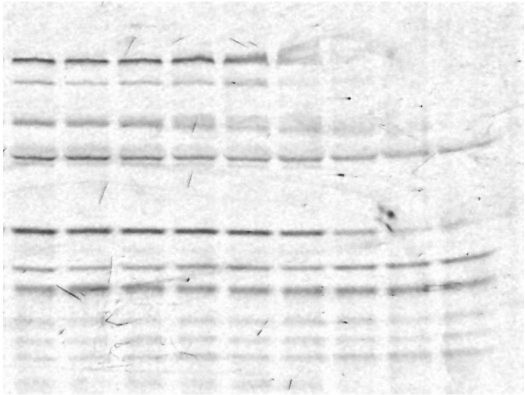

Replicate 2 – original gel image

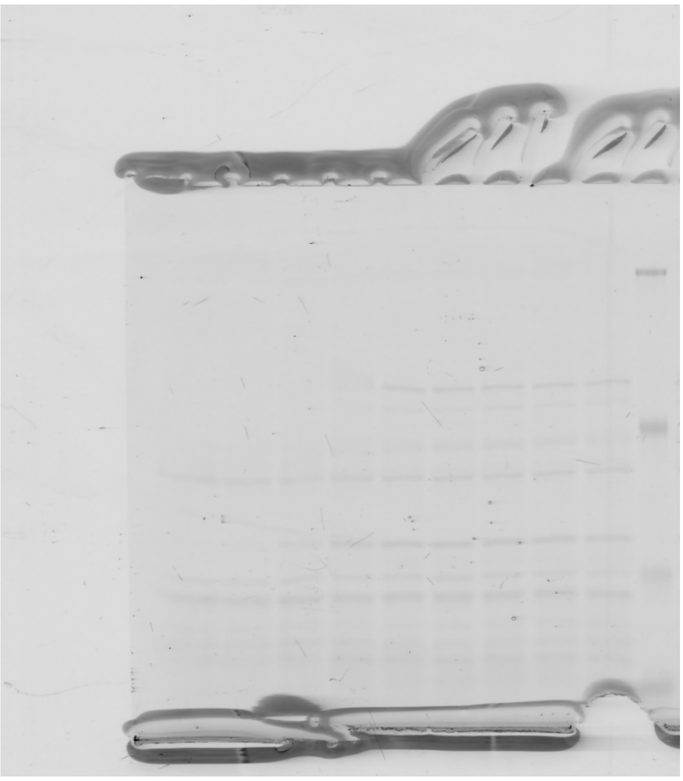

Replicate 2 - Adjusted image used for calculation of PBP inhibition

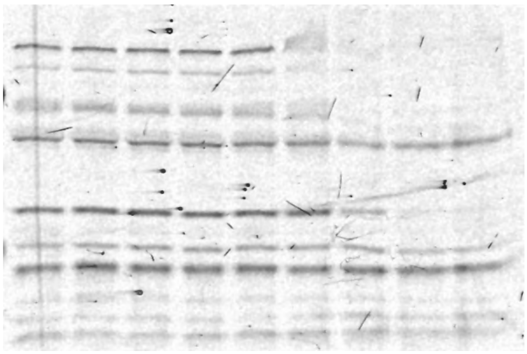

Ceftazadime titration

Replicate 1 – original gel image

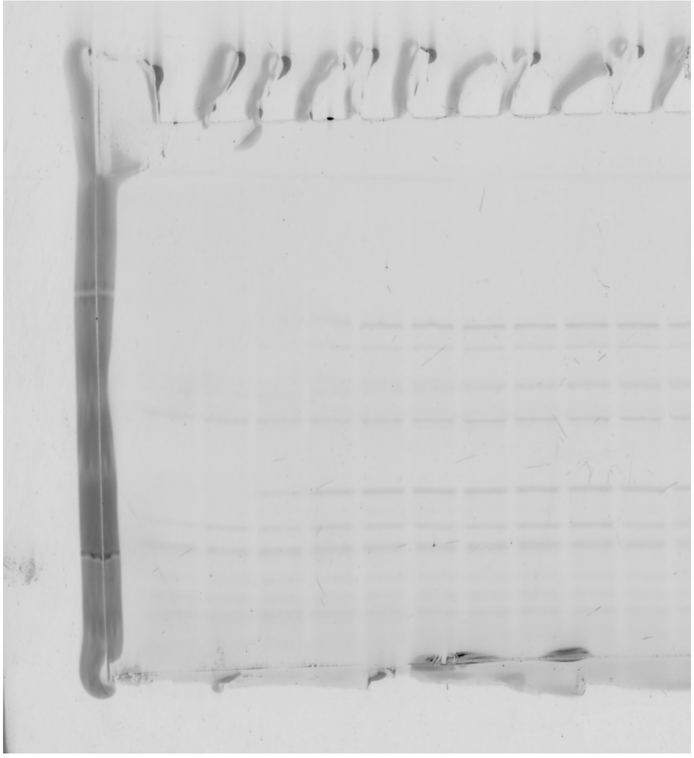

Replicate 1 – adjusted image used for calculation of PBP inhibition

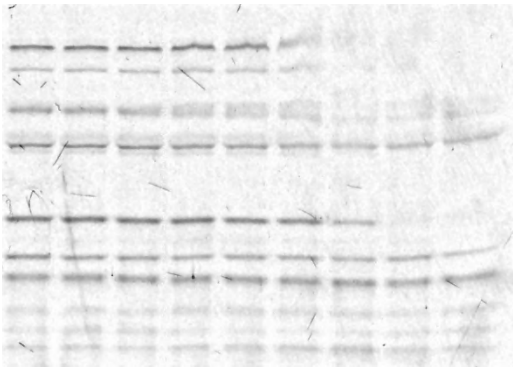

Replicate 2 – original gel image

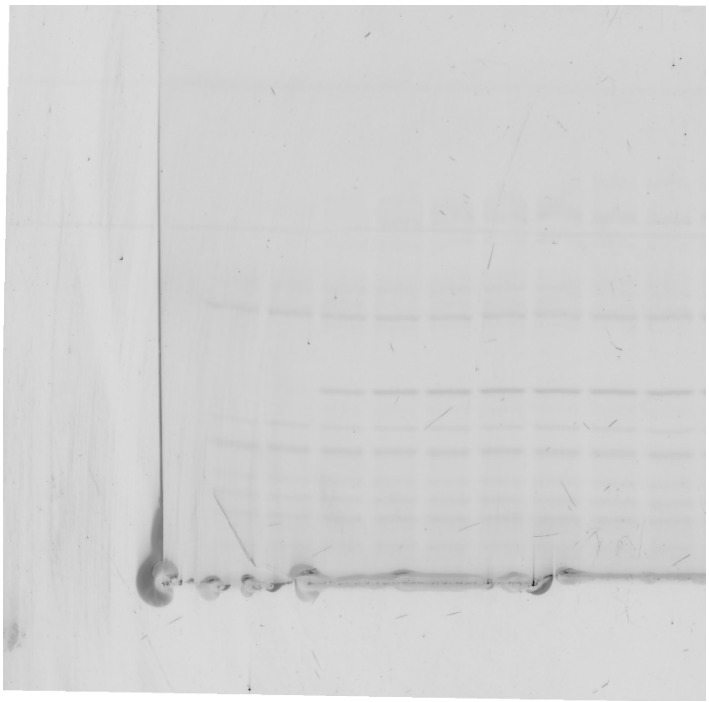

Replicate 2 – adjusted image used for calculation of PBP inhibition

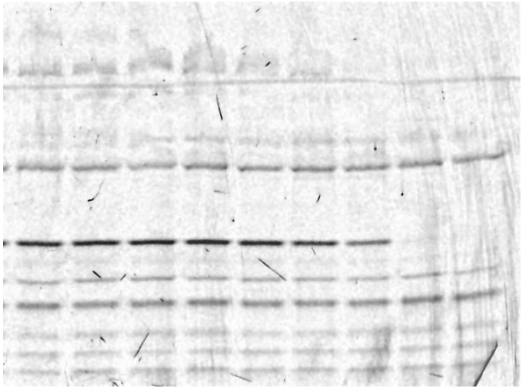

<u>Meropenem titration</u>

Replicate 1 – original gel image

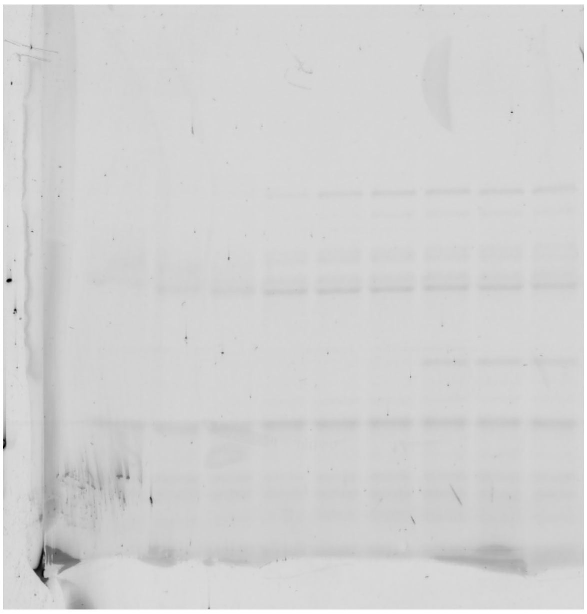

Replicate 1 – adjusted image used for calculation of PBP inhibition

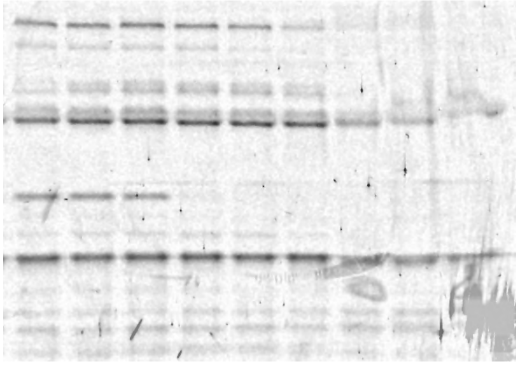

Replicate 2 – original gel image

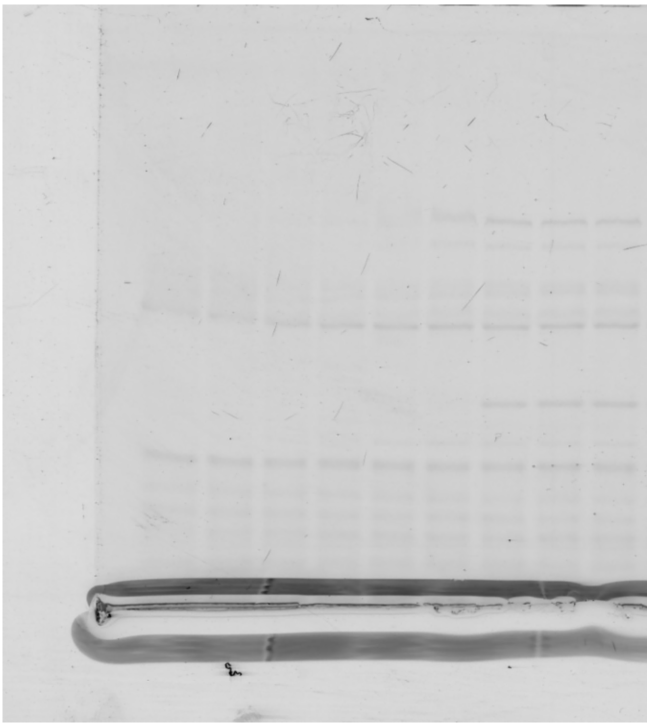

Replicate 2 – adjusted image used for calculation of PBP inhibition

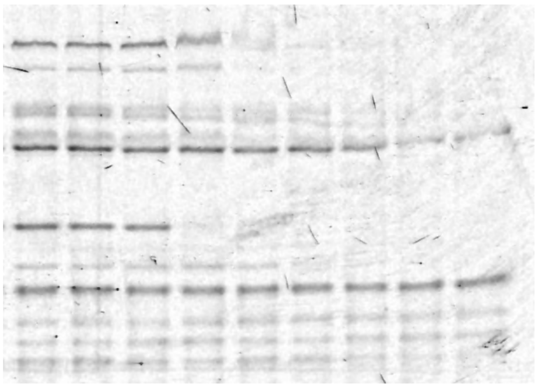

<u>Aztreonam + meropenem combination titration</u>

Replicate 1 – original gel image

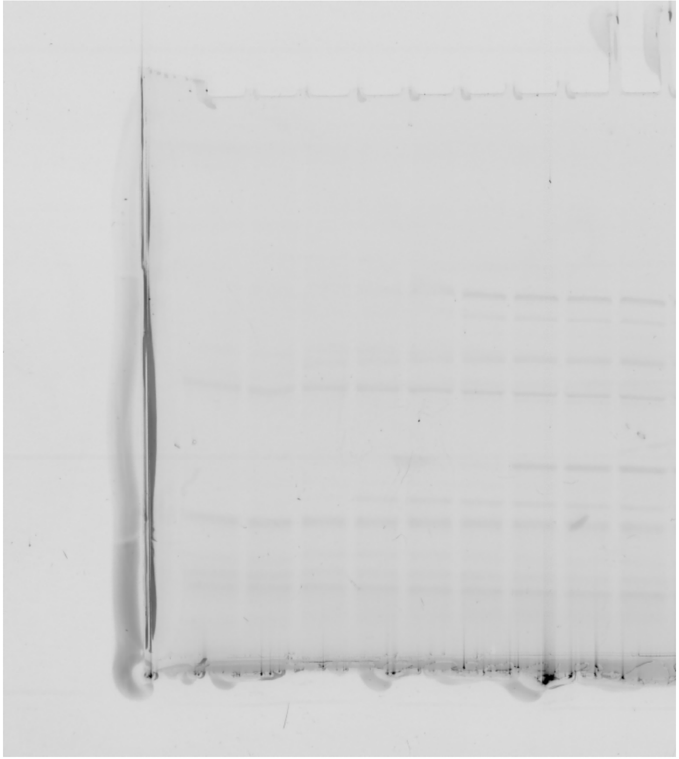

Replicate 1 – adjusted image used for calculation of PBP inhibition

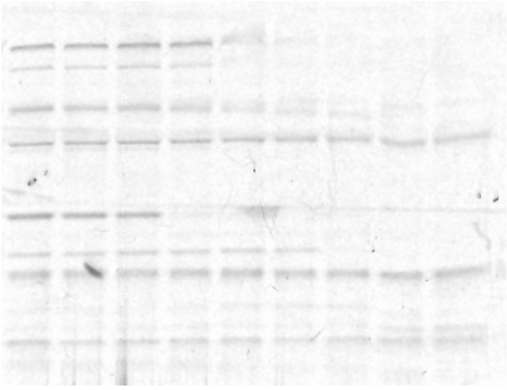

Replicate 2 – original gel image

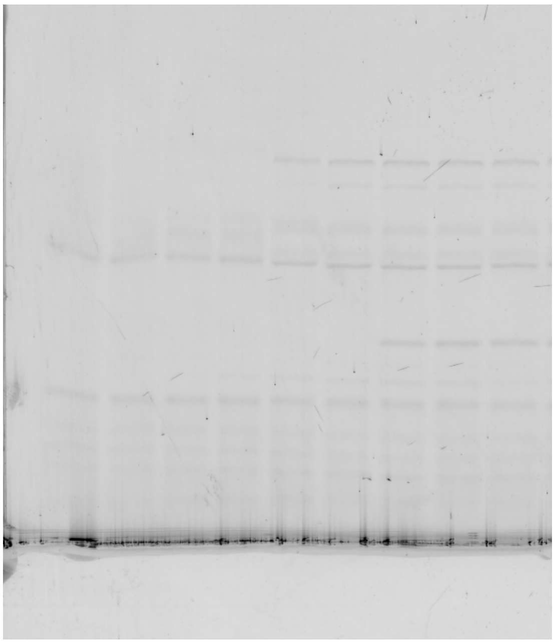

Replicate 2 – adjusted image used for calculation of PBP inhibition

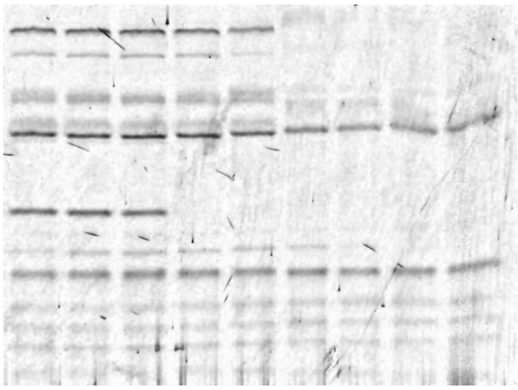

<u>Ceftazidime + meropenem combination titration</u>

Replicate 1 – original gel image

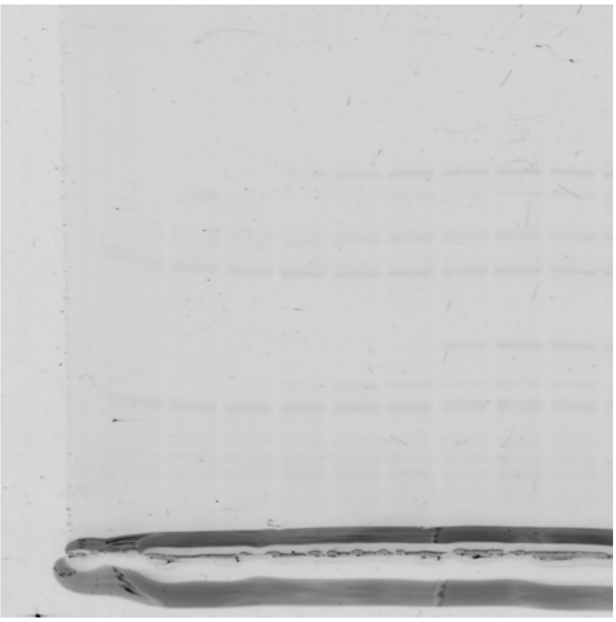

Replicate 1 – adjusted image used for calculation of PBP inhibition

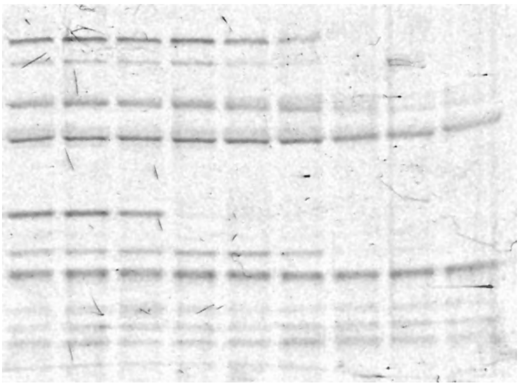

Replicate 2 – original gel image

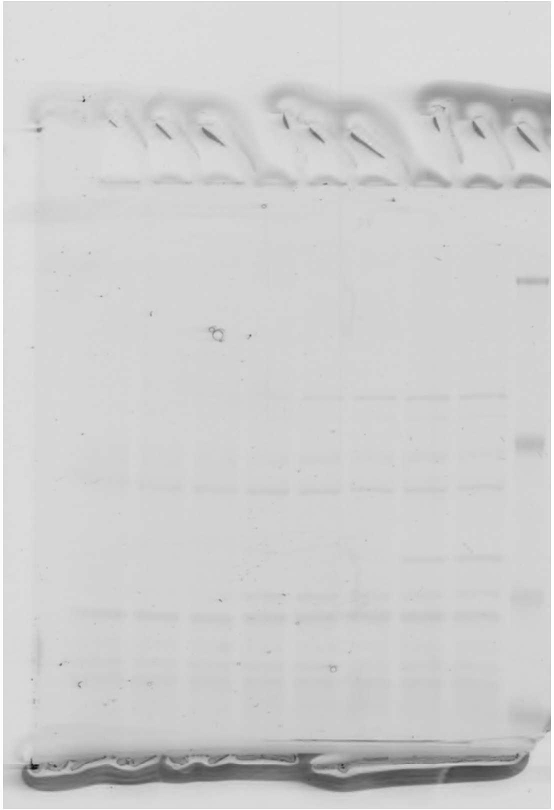

Replicate 2 – adjusted image used for calculation of PBP inhibition

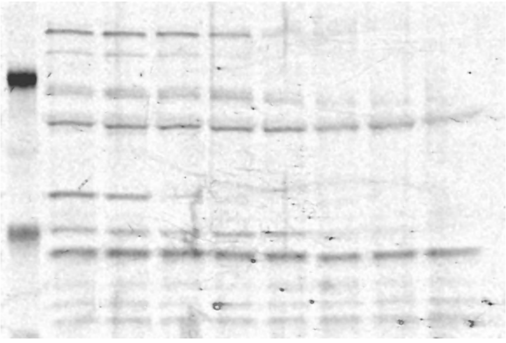

Supplement: Supplementary file 1 — Supplementary Information [file 41598_2019_45550_MOESM1_ESM.pdf]
